# In-depth characterization of *Klebsiella pneumoniae* carbapenemase (KPC)-encoding plasmids points at transposon-related transmission of resistance genes

**DOI:** 10.3389/fcimb.2025.1542828

**Published:** 2025-03-13

**Authors:** Vincent F. van Almsick, Annika Sobkowiak, Natalie Scherff, Franziska Schuler, Johannes Benedict Oehm, Christian Böing, Alexander Mellmann, Vera Schwierzeck

**Affiliations:** ^1^ Institute of Hygiene, University Hospital Münster, Münster, Germany; ^2^ Department of Cardiology I – Coronary and Peripheral Vascular Disease, Heart Failure, University Hospital Münster, Münster, Germany; ^3^ Institute of Medical Microbiology, University Hospital Münster, Münster, Germany; ^4^ Institute of Medical Informatics, University of Münster, Münster, Germany

**Keywords:** antimicrobial resistance, KPC-3, long-read whole genome sequencing, *Klebsiella pneumoniae*, hospital transmission, transposon

## Abstract

Antimicrobial resistance (AMR) is a growing threat in healthcare systems, particularly in the management of infections in critically ill patients. This study highlights how to identify clusters and putative sharing of mobile genetic elements, such as transposons, in the hospital setting using long-read whole genome sequencing (lrWGS). The approach described here can be employed to investigate the transmission dynamics of KPC-3-positive *Klebsiella pneumoniae* at multiple levels, from the entire isolate down to individual plasmids and transposons. Here, a *bla*_KPC-3_ harboring transposon cluster was identified by using a Mash-based distance calculation for plasmids. This approach was used to investigate a local accumulation of KPC-3-positive *Klebsiella pneumoniae* on surgical and infectious disease wards of a tertiary care center in Germany over a time of six months. In total, seven patients were affected. Core genome multi-locus sequence typing analysis (cgMLST) identified two distinct genetic clusters: a sequence type (ST) 307 cluster (n = 5) and a ST101 cluster (n = 2). All isolates carried a *bla*_KPC-3_ carbapenemase. Further Mash distance-based plasmid analysis was not consistent with plasmid transfer due to genetic heterogeneity, but identified a transposon cluster across all isolates. Infection control evaluation of patient movements within their hospital admission supports a possible clonal transmission. Subsequent infection control measures, including point prevalence screening and enhanced contact precautions, successfully contained further transmissions. The study illustrates the value of in-depth plasmid analysis in understanding the transmission dynamics and epidemiology of AMR, particularly in hospital environments.

## Introduction

Antimicrobial resistance (AMR) has emerged as a significant challenge in healthcare settings. This issue is especially pronounced in the management of infections among critically ill or immunocompromised patients (van Duin and Doi, 2017; Antimicrobial Resistance Collaborators, G, 2022). While extensive research has been conducted on AMR genes, the role of mobile genetic elements (MGE) like AMR plasmids or transposons and their horizontal transfer, particularly in nosocomial settings and outbreak investigations, has not been widely studied (Gilchrist et al., 2015; Rose et al., 2023). Recent advancements in long-read whole genome sequencing (lrWGS) technologies now allow diagnostic laboratories to incorporate comprehensive analyses of mobile genetic elements into outbreak management and routine molecular surveillance (Gilchrist et al., 2015; van Almsick et al., 2022; Agustinho et al., 2024). *Klebsiella pneumoniae*, a commensal Gram-negative bacterium, has become a prevalent pathogen in healthcare environments (Martin and Bachman, 2018; Agustinho et al., 2024). For many years, one common treatment option for extended-spectrum beta-lactamase producing *K. pneumoniae* was carbapenems. Recently, the number of Klebsiella pneumoniae carbapenemase (KPC)-positive *K. pneumoniae* isolates has been increasing worldwide since the first identification of KPC in 1996 in the USA (Yigit et al., 2001). This is posing serious challenges to treatment options and complicating therapeutic approaches. In Germany, however, there is a relatively low overall incidence of KPC, while the predominant carbapenemase detected is *bla*_OXA-48_.

KPC enzymes have the ability to hydrolyze all beta-lactam antibiotics, and the co-existence of other AMR genes worsens the scenario, classifying these organisms as multidrug-resistant (MDRO) and limiting treatment options even further. KPC genes are frequently associated with MGEs (Yang et al., 2021), which facilitate horizontal gene transfer (HGT) among bacterial populations and strains, significantly contributing to the spread of resistance (Munoz-Price et al., 2010; Rojas et al., 2017). Several KPC-3 outbreaks have been previously reported in regions and hospitals (Stillwell et al., 2015; Perdigao et al., 2019; Soria-Segarra et al., 2020).

This report investigates a local transmission event of a KPC-3 gene associated with a transposon in *K. pneumoniae* within a tertiary hospital, emphasizing the implications of plasmid and mobile genetic element analysis for effective outbreak management and molecular surveillance.

## Methods

### Microbiological methods

The seven collected isolates were cultivated and identified according to standardized microbiology methods described here (Sobkowiak et al., 2024). All screening samples were collected as part of the routine hospital surveillance of multi-drug-resistant bacteria (MDRB) according to national recommendations. Screening specimens were cultivated on chromID ESBL Agar (bioMérieux, Marcy-l’Étoile, France) and incubated at 36°C ± 1°C for 18 to 24 h. Clinical specimens were cultivated on Columbia Blood agar with 5% sheep blood (BD, Heidelberg, Germany; aerobic incubation at 35°C ± 2°C ambient air for up to 3 days) and MacConkey selective agar for Gram-negative bacteria (MacConkey II Agar, BD, Heidelberg, Germany; aerobic incubation at ambient air 35°C ± 2°C for up to 2 days), if applicable. Environmental sampling was performed using polywipes (mwe, Corsham, Wiltshire, UK) on contact surfaces and incubating them in Tryptic Soy Broth containing lecithin tween (LT) (Merck Millipore, Eppelheim, Germany) for 24 h at 37°C. The broth was streaked out onto Columbia blood agar (Thermo Scientific, Schwerte, Germany).

For all isolates, species identification was performed by matrix-assisted laser desorption/ionization time-of-flight mass spectrometry (MALDI-TOF/MS) (MALDI-TOF MS, Biotyper^®^ Sirius one, Bruker, Bremen, Germany) with scores above 2.0. Antimicrobial susceptibility testing was performed using a Vitek2^®^ automated system (bioMérieux, Marcy l’Étoile, France) applying EUCAST clinical breakpoints 2024. Carbapenem resistance was validated using E-Test (bioMérieux) and standard PCR methods (eazyplex^®^SuperBug CRE, amplex, Gars-Bahnhof, Germany) confirmed the presence of a *bla*_KPC-3_ carbapenemase gene.

### DNA extraction and long-read whole genome sequencing

Genomic DNA of all isolates was extracted using the ZymoBIOMICS 96 MagBead DNA Kit (Zymo Research, Irvine, CA, USA). Subsequent sequencing was performed on a PacBio^®^ Sequel IIe system using the SMRTbell^®^ Express Template Prep Kit 2.0 (Pacific Biosciences Inc., Menlo Park, CA, USA) as described before (Effelsberg et al., 2021). Sequencing data was assembled *de novo* using the SMRT^®^ Link software suite 11 (Pacific Biosciences Inc.) with default parameters.

All bioinformatic analyses were performed using SeqSphere+ software version v10 (Ridom GmbH, Münster, Germany) with NCBI AMRFinderPlus v3.11.26 (Feldgarden et al., 2019) and the long-read data plasmid transmission analysis module (Scherff et al., 2024).

For backwards compatibility, the multilocus sequence typing (MLST) sequence types (STs) were also extracted from the WGS data. Phylogenetic trees were constructed based on cgMLST using a neighbor-joining approach. Here the standardized cgMLST scheme for *Klebsiella pneumoniae* was used. For plasmid reconstruction and subsequent typing, different modules from the MOB-suite v3.1.8 software (Robertson and Nash, 2018) implemented in SeqSphere^+^ were used. Circular contigs shorter than 500 kb were automatically regarded as plasmids; contigs of fragmented plasmids were reconstructed using the MOB-recon module. Based on plasmid sequences, we constructed a Mash database with a k-mer size of 21 nucleotides and a sketch size of 10,000. For distance calculation, we additionally included a size correction, where for each 1% size difference of the plasmids, the corrected Mash distance was lowered by 0.0003 to compensate for insertions or deletions of larger repetitive sequence fragments. For plasmids with a size difference of more than 40%, the uncorrected value was kept to account for possible multimer formations. Plasmids with a (corrected) Mash distance of ≤ 0.001 were regarded as highly similar, suggesting a potential transmission.

Plasmids were further characterized on the proksee (Grant et al., 2023) platform with a BLAST (Camacho et al., 2009) comparison. Subsequent annotation was performed using Bakta (Schwengers et al., 2021) and Dfast (Tanizawa et al., 2018) and manually curated. SnapGeneViewer (v. 7.2.0) (http://www.snapgene.com/) was used to visualize the transposon. The SNP analysis was performed by alignments utilizing MEGA v11.0.9 (Tamura et al., 2021).

All isolates are submitted under the NCBI bioproject accession number PRJNA1186135.

Plasmids were compared to publicly available sequence data using BLASTn against the NCBI database and a Mash comparison against PLSDB (Schmartz et al., 2022), a database containing plasmid sequences only.

### Epidemiological evaluation of potential transmission events

Potential transmission events were evaluated based on patients′ medical records as well as nursing and medical staff interviews by trained infection prevention and control staff. A hospital contact suggesting a nosocomial transmission was defined as a contact on room or ward level if the patient was admitted to the same room or ward for at least one overlapping calendar day within the hospital stay. Identification of a KPC harboring *Klebsiella pneumoniae >*48h after admission to the hospital was defined as nosocomial.

## Results

### Characteristics of transmission event

**Figure 1:**
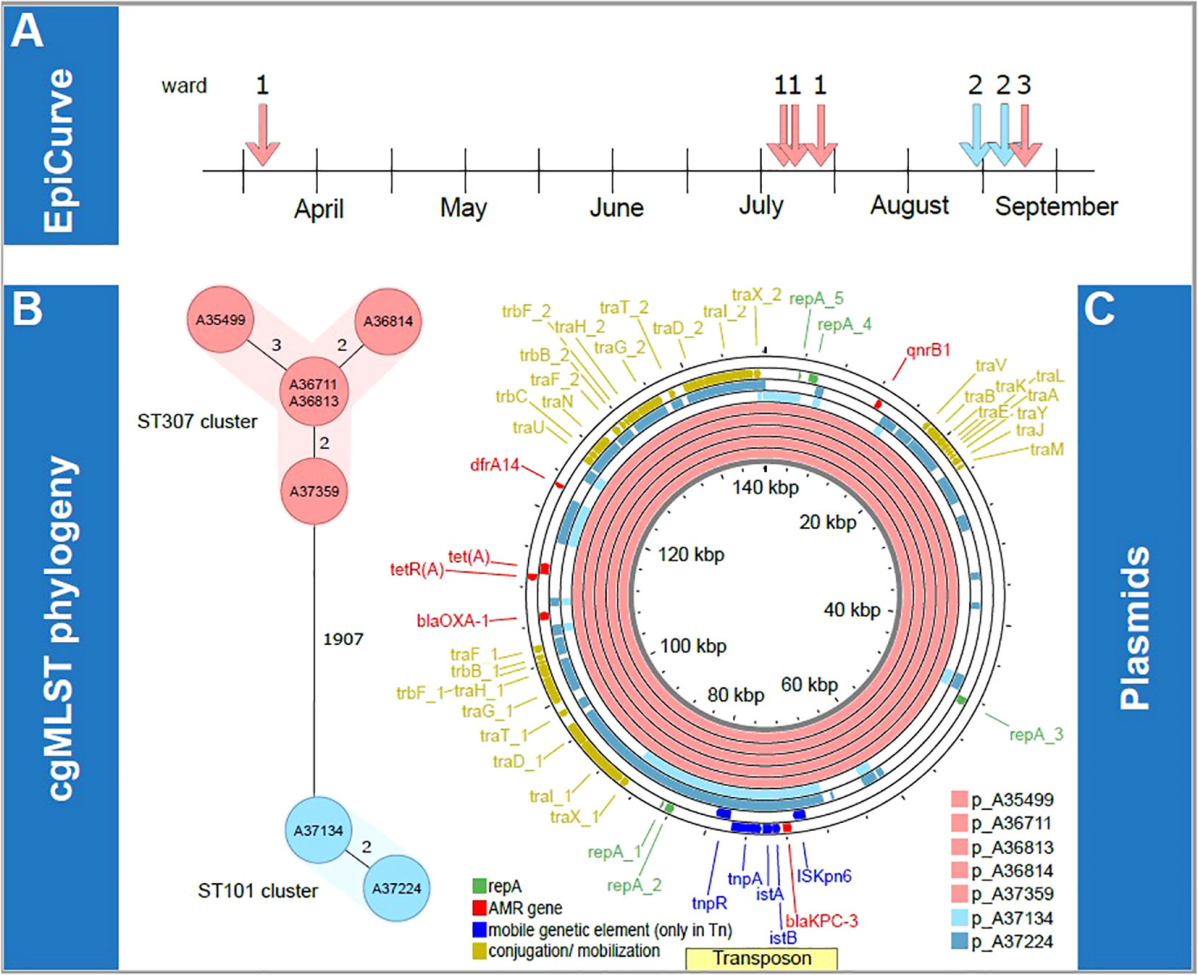
Overview of transmission clusters and underlying plasmid characterization. First, epidemiological information concerning the isolates is illustrated. The epiCurve represents the time the isolates were identified and the ward where the patients were treated **(A)**. Next, genetic analysis of all isolates is shown. cgMLST phylogeny **(B)** and respective plasmids of all isolates **(C)** are visualized in the lower panel. Here the color is given according to the associated ST clusters (light red: ST307 cluster, light blue: ST101 cluster). AMR, antimicrobial resistance; kbp, kilobase pairs; p, plasmid; ST, sequence type.

We identified two clusters of KPC-3-positive *K. pneumoniae* on two surgical wards (ward 1 and ward 2) and on one infectious disease ward (ward 3) at a 1450-bed tertiary care center in Münster, Germany, over a six-month period (March to August 2024; see **Figure 1A**). All isolates displayed resistance to carbapenems, quinolones, acylaminopenicillins, and third-generation cephalosporins. Seven patients were affected with a median age of 48 years (IQR 38-57), including six males and one female. One patient was transferred from Ukraine to receive treatment for war-related injuries; all the other patients have received their general care in our region.

### Molecular data analysis

Molecular analysis using lrWGS assigned the isolates to two different sequence types (ST) 307 (n=5) and ST101 (n=2), which formed two clearly distinct clonal clusters that differed in ≥ 1907 cgMLST targets. Within the two clusters, cgMLST revealed a maximum distance of three alleles between isolates, suggesting a high degree of genetic similarity (**Figure 1B**). Epidemiological investigations supported the possibility of clonal transmission for isolates of the ST307 cluster on ward 1 until July, as the four patients shared hospital rooms or wards during their most recent hospital admission (**Table 1**). One further probable transmission of that cluster occurred in late August on the infectious disease ward after one colonized patient was transferred to that ward. One patient was tested positive for KPC-3-positive *K. pneumoniae* while occupying a room formerly inhabited by two colonized patients. Here, insufficient cleaning of surfaces in the patients’ room could have been a contributing factor for transmission. The patients from the ST101 cluster both shared a hospital room on ward 2 but had no epidemiological link with patients of the ST307 cluster on ward 1. No previous contact between patients within the hospital was identified based on the analysis of electronic patient records looking back to January 2022.

**Table 1:** Characteristics of isolates and epidemiological information.

Isolate	Isolation date	Material	ST	Nosocomial	Contact tracing	
					Room contact	Ward contact
A35499	03-20-2024	screening swab	307	yes		Index A37611 (4 days)
A36814	07-05-2024	screening swab	307	yes	A36711 (1.67 days)	
A36711	07-08-2024	Blood culture (central line)	307	yes	see above	
A36813	07-12-2024	Screening swab	307	yes		A36711 (2 days) former room of A36814/A36813
A37134	08-14-2024	Screening swab	101	yes	A37224 (7h)	
A37224	08-19-2024	Screening swab	101	yes	see above	
A37359	08-23-2024	Intraoperative swab (pararectal abscess)	307	yes		A36711 (6 days)

ST307 cluster, light red; ST101 cluster, light blue.

**Figure 2:**
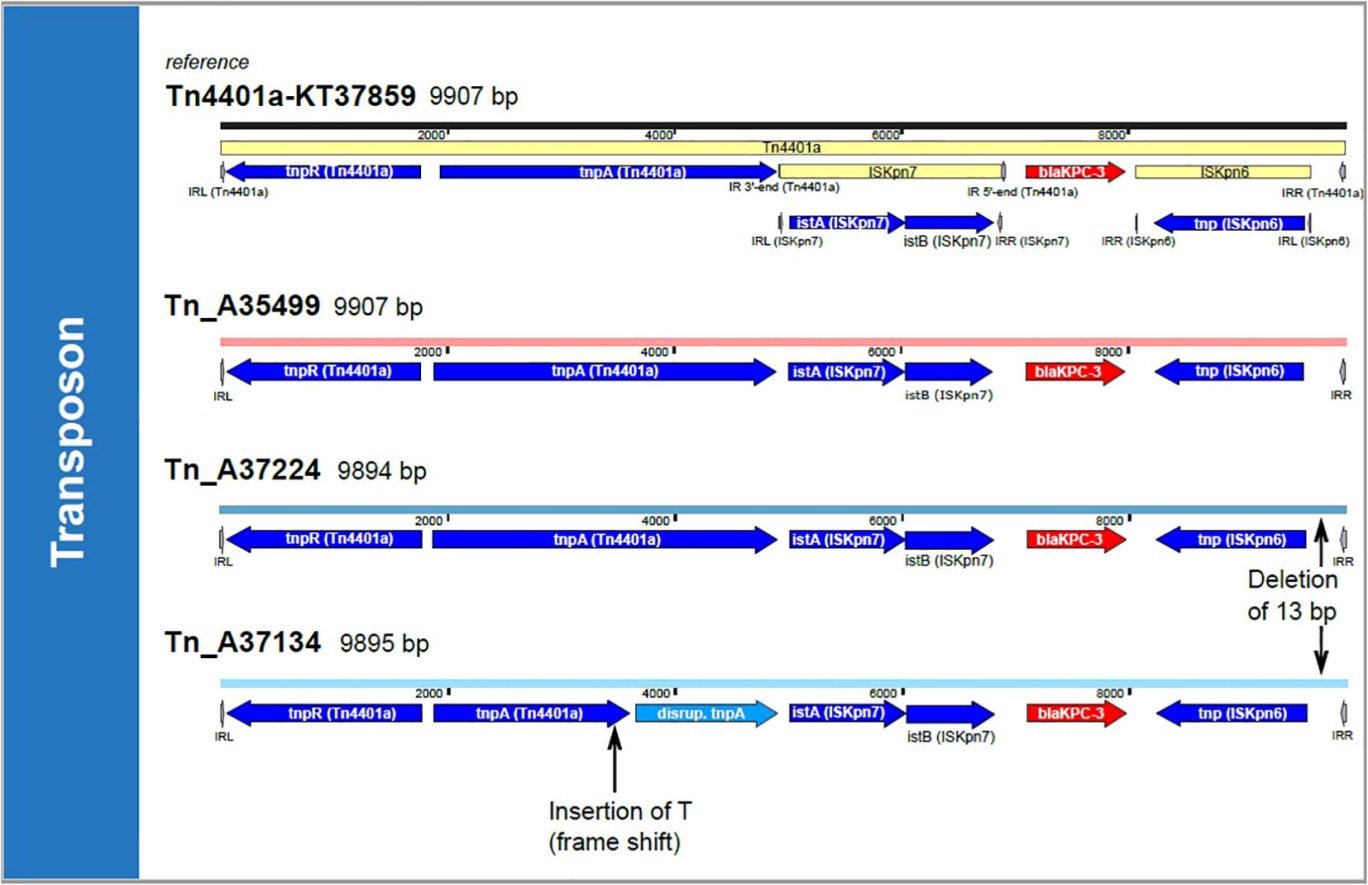
Transposon comparison. The transposon Tn4401a-KT37859 is shown on top of the figure. Colors of the associated harboring plasmid represent the ST clusters (light red: ST307 cluster, light blue: ST101 cluster). The transposons were compared by a BLASTn approach and SNP analysis. All transposons in cluster ST307 are 100% identical with 100% coverage; therefore, only one example, Tn_A35499, is shown. Tn, Transposon; T, Thymine; bp, base pairs.

Next, we aimed to determine whether the two clusters (ST307 and ST101) shared the same plasmids. lrWGS confirmed that in all cases, *bla*_KPC-3_ was localized on an IncF plasmid (see **Figure 1C**). Mash distance was used to estimate the genetic similarity of plasmids (Scherff et al., 2024). KPC-3-positive plasmids in the ST307 cluster show high similarity (from 0 to 0.0001) in the Mash distances, offering further evidence for the putative clonality of the isolates. The KPC-3-positive plasmids in the ST101 cluster in comparison did not show that high degree of similarity (Mash distance 0.0402) but still remained with the IncF type. Plasmid comparison using Mash distance and BLASTn against the NCBI database failed to identify any plasmids with comparable levels of similarity beyond the clonal ST307 clusters (KPC-3-positive plasmid cluster best hit: accession number ON081620.1 with query coverage of 78% and 100% percent identity). In the ST101 cluster, we found a similar KPC-3 positive plasmid (acc. no. MT809698.1 with a query coverage of 100% and 99.95% percent identity). Similar results were found in the PLSDB. Comparable plasmids in the PLSDB with a Mash distance threshold of 0.005 were only found for p_A37134, i.e., NZ_MZ606382.1 and NZ_MZ606380.1. For all other blaKPC-3 harboring plasmids, the first hits were found with a threshold of 0.01, indicating structural differences and a low degree of similarity (**Supplementary Table 2**). A comparison of *bla*_KPC-3_-harboring plasmids from both clusters using Mash distance did not suggest plasmid transmission between the two clusters due to significant genetic differences of the plasmids. The minimal Mash distance detected was 0.0613 and 0.0681, calculated from both KPC-3 positive plasmids from the ST101 cluster to the ST307 KPC-3 positive plasmid cluster. However, we did identify a nearly identical region in all *bla*_KPC-3_-positive plasmids that includes the *bla*_KPC-3_ gene. This observation is consistent with the possible transmission by conjugation of small mobile genetic elements at an unknown time. In a comparison with the transposon-database TnCentral (Ross et al., 2021), which is based on a BLAST comparison, we found a similar transposon (Tn4401a-KT378596, 9907 kb) with a query cover of 100% and >99% identity (i.e. one or two single nucleotide polymorphisms, see **Figure 2**). All our isolates have the SNP T9783G in comparison to the reference. SNP analysis of the potential transposon shared among the isolates, confirmed the genetic similarities of this region in all isolates.

### Infection control measures

Point prevalence screening on ward 1 did not identify any unknown carriers of KPC-3 positive *K. pneumoniae* isolates. Enhanced contact precautions were implemented for all patients identified as *bla*_KPC-3_ carriers as per local guideline for all patients with carbapenemase-producing Gram-negative bacteria. Environmental sampling of high-touch surfaces and medical devices was carried out on the affected wards. In total, 25 environmental samples were taken. 9 samples from high-touch surfaces of patient rooms, including the patients’ bathrooms. 2 samples were taken from frequently used medical devices on the ward (ultrasound and vital sign monitor). The remaining 14 samples were taken from surfaces on the ward, including work surfaces exclusively frequented by healthcare workers, such as the area dedicated to the preparation of IV medication. Samples gave no indication of the presence of *K. pneumoniae* on surfaces. Extensive disinfection measures were performed in all patient rooms as well as in the medication preparation area and storage rooms of the affected wards. In addition, the medical staff received intensified training in hygiene practices from hygiene specialists. Until the day of writing, no further new nosocomial cases of *bla*_KPC-3_ carriers were identified on the affected wards following the last case in August 2024.

## Discussion and conclusions

In this report, we documented two independent clusters of KPC-3 positive *K. pneumoniae* on two surgical wards and one infectious disease ward of a tertiary care hospital, affecting seven patients over a six-month period. lrWGS-based analysis could clearly discriminate two distinct cgMLST clusters, which were further verified by both in-depth plasmid analyses that revealed high sequence similarity in the plasmid backbones in the ST307 cluster, and epidemiological contact tracing. In our setting, a hospital with a low incidence of carbapenemase-producing MDRO, WGS is helpful to distinguish whether cases are mere “coincidence” or have to be regarded as possible transmission. Once cases have been identified as possible transmission, WGS data can be helpful to illustrate transmission pathways as part of staff education and to justify the implementation of infection control measures such as screening and contact precautions. While classical surveillance data usually only document resistance patterns, molecular surveillance by WGS helps to investigate evolutionary pathways and regional distribution of resistance genes.

*K. pneumoniae*, and especially ST307, is known to cause outbreaks in healthcare facilities, which increased in the European Union since the Russian invasion of Ukraine (Sandfort et al., 2022; Stein et al., 2023); a contributing cause may be the increased number of foreign patients in various European healthcare centers for specific and complex medical treatments due to war injuries (Zwittink et al., 2022; Ljungquist et al., 2023).

Plasmid analysis revealed that the *bla*_KPC-3_ gene was located on an IncF plasmid in all cases. Despite the shared presence of *bla*_KPC-3_, plasmid comparison between ST307 and ST101 clusters showed significant genetic differences, suggesting that plasmid transmission between these clusters was unlikely. The absence of similar plasmids compared to the ST307 KPC-3 positive plasmids in the NCBI database and the PLSDB indicates that the plasmids involved in this investigation are rare or less commonly encountered and may represent a recently emerged variant. The presence of an identical region across all plasmids, including the *bla*_KPC-3_ gene, suggests the role of mobile genetic elements, in this case of transposons, in horizontal gene transfer (Sheppard et al., 2016). While the horizontal gene transfer cannot be definitively proven based on the available data, it remains a plausible explanation for the spread of the *bla*_KPC-3_ gene within the hospital environment. The association of *bla*_KPC-3_ with this mobile genetic element might offer an explanation why the incidence of *bla*_KPC-3_ is rising (Budia-Silva et al., 2024). This emphasizes the importance of establishing a continuous surveillance and characterization of AMR plasmid content in clinical isolates.

lrWGS has become a key technology to identify plasmids and mobile genetic elements carrying AMR across different MDROs. This study is a single case report only but the study demonstrates the value of modern sequencing technology during MDRO surveillance. lrWGS with plasmid analyses can provide further information, rule out plasmid transmissions, and improve our understanding of the transmission of AMR in hospitals. Mobile genetic elements have a significant impact on the spread of AMR in hospital settings (Evans et al., 2020). Yet, due to the shortfalls of short-read sequencing technology, our knowledge of the transmission of plasmids and mobile genetic elements in the context of hospitals is limited. Transmission(s) via mobile genetic elements are a plausible explanation for the spread of the *bla*_KPC-3_ gene; however, further research on causality, specific transmission pathways, and underlying mechanisms is necessary.

## Data Availability

The datasets presented in this study can be found in online repositories. The names of the repository/repositories and accession number(s) can be found below: https://www.ncbi.nlm.nih.gov/, PRJNA1186135.
