# Supplementary material for: In-depth characterization of Klebsiella pneumoniae carbapenemase (KPC)-encoding plasmids points at transposon-related transmission of resistance genes
Source: Front Cell Infect Microbiol. 2025 Mar 13;15:1542828. doi: 10.3389/fcimb.2025.1542828 (PMC11948313; doi:10.3389/fcimb.2025.1542828)
Supplement: Supplementary file 1 [file DataSheet1.pdf]

Supplement to:

In-depth characterization of *Klebsiella pneumoniae* carbapenemase (KPC)-encoding plasmids points at transposon-related transmission of resistance genes

Supp. Table 1: Information on sequencing metrics

Isolate	Isolation date	Average Coverage	Contigs	GC content	Percentage of identified cgMLST targets	Plasmid count
A35499	03-20-2024	45	4	57.2	99.1	3
A36814	07-05-2024	18	5	57.2	98.5	4
A36711	07-08-2024	30	4	57.2	98.8	3
A36813	07-12-2024	42	4	57.2	98.7	3
A37134	08-14-2024	12	14	56.5	97.3	6
A37224	08-19-2024	14	8	56.5	98.2	5
A37359	08-23-2024	18	4	57.2	98.5	3

Supp. Table 2: Number of hits (and accession numbers, if applicable, and number of hits <10) for a Mash distance search for different maximum distance thresholds in the PLSDB v.2024_05_31_v2

isolates	0.001	0.005	0.1
p_A35499	0	0	44
p_A36814	0	0	43
p_A36711	0	0	43
p_A36813	0	0	3 (CP067581.1; CP067645.1; NZ_KU665642.1)
p_A37134	0	2 (NZ_MZ606382.1; NZ_MZ606380.1)	4 (NZ_MZ606382.1; NZ_MZ606380.1; NZ_CP110970.1; NZ_CP110778.1)
p_A37224	0	0	1 (NZ_CP033627.1)
p_A37359	0	0	44
